# Application of Human Papillomavirus‐DNA Test on Salivary Gland Fine Needle Aspiration Cytology Samples Confirms the Absence of the Virus in Primary Neoplasms and Demonstrates for the First Time Its Presence in Salivary Intraglandular Cysts

**DOI:** 10.1002/dc.25425

**Published:** 2024-12-10

**Authors:** Immacolata Cozzolino, Andrea Ronchi, Marco Montella, Raffaella Ruggiero, Rosaria Cappiello, Giovanni Savarese, Giuseppe Colella, Renato Franco

**Affiliations:** ^1^ Pathology Unit, Department of Mental and Physical Health and Preventive Medicine University of Campania Luigi Vanvitelli Naples Italy; ^2^ AMES‐Centro Polidiagnostico Strumentale, SRL Naples Italy; ^3^ Maxillo‐Facial Surgery Unit, Multidisciplinary Department of Medical, Surgical and Dental Specialty University of Campania Luigi Vanvitelli Naples Italy

**Keywords:** cysts, fine needle aspiration cytology, human papillomavirus, neoplasms, salivary gland

## Abstract

**Background:**

The correlation between Human Papillomavirus (HPV) and salivary gland neoplasms is still controversial. Data in the literature are conflicting, reporting the presence of the HPV‐DNA in a significant percentage of cases or none. We investigated the presence of HPV in a series of salivary gland neoplasms using fine needle aspiration cytology (FNAC) samples to explore its potential oncogenic role in salivary gland tumor development.

**Methods:**

The study included 66 salivary gland lesions from 65 patients. For all cases, residual cytological material in the needle hub, once direct smears were obtained, was fixed by rinsing the needle in Cytolyt hemolytic and preservative solution (Hologic Inc. Marlborough, MA 01752 USA). The samples fixed in the Cytolyt hemolytic and preservative solution (Hologic Inc. Marlborough, MA 01752 USA) were centrifuged at 252 *g*, then the sediment was transferred to the Preservcyt solution and used for the detection of HPV‐DNA.

**Results:**

We found evidence of the presence of HPV in two salivary intraglandular cysts lined by squamous cell epithelium. Furthermore, we also found HPV in three metastatic oropharyngeal squamous cell carcinomas (SCC) located in the parotid gland. Regarding primary salivary gland tumors, the HPV test produced negative results in all cases.

**Conclusion:**

HPV testing produced negative results in all primary salivary gland tumors, failing to confirm the potential involvement of HPV infection in the pathogenesis of salivary gland tumors. Instead, the presence of HPV DNA in samples of salivary intraglandular cysts, never reported before, may be an interesting fact, which however requires further evaluation.

## 1 Introduction

The pathogenesis of salivary gland tumors remains elusive, with no specific known causative factors identified. Over the past 15 years, conflicting evidence has emerged regarding the potential involvement of human papillomavirus (HPV) infection in the development of salivary gland tumors. While the correlation between HPV and carcinomas of the oral cavity and oropharynx in the head and neck region is now well‐established, uncertainty persists regarding the presence of the virus within salivary glands and its potential role in tumor development.

Initial findings by Vageli et al. reported the presence of the HPV viral genome in a significant percentage of cases [1]. However, subsequent studies failed to confirm this correlation, thereby leaving the issue unresolved [1, 2, 3]. Furthermore, there is a need for further clarification on the clinical and biological implications of evidence suggesting the presence of HPV in the saliva of a certain percentage of individual [1, 4, 5].

It is currently understood that HPV tends to reside in tonsillar crypts, exploiting existing biofilms. However, the virus obstacles when replicating, struggling to release newly formed virions into the surrounding environment, which leads to their entrapment within the biofilm. This not only facilitates the establishment of an infection reservoir but also enables the virus to persist in a latent state by exploiting immune evasion mechanisms [6].

Based on these findings, we investigated the presence of HPV in a series of salivary gland neoplasms using fine needle aspiration cytology (FNAC) to explore its potential oncogenic role in salivary gland tumor development.

## 2 Materials and Methods

### 2.1 Cases Selection

The study included 66 cases of salivary gland lesions from 65 patients enrolled between May 2022 and January 2023. Each patient underwent ultrasound (US)‐FNAC for salivary gland nodules at the FNAC outpatient facility of AOU “L. Vanvitelli.” During the FNAC procedure, patients were informed about the diagnostic process and associated risks. Additionally, written informed consent, including permission to utilize the diagnostic data for scientific purposes, was obtained from each patient. The study was conducted according to the guidelines of the Declaration of Helsinki and approved by the ethics committees of AOU “L. Vanvitelli” and AOPN “Ospedale dei Colli” (protocol code 5901; approval date April 21, 2021).

### 2.2 Samples Management

All US‐guided FNACs of salivary gland lesions were performed by an interventional cytopathologist using a systematic approach. The procedure began with US evaluation of the salivary gland region to identify the nodule sites. Subsequently, FNACs of the salivary gland nodules were performed according to standard procedure. The first pass cytological material was used to prepare air‐dried and alcohol‐fixed direct smears. The residual material in the hub of the first‐pass needle was fixed by rinsing the needle in the hemolytic and preservative solution Cytolyt (Hologic Inc. Marlborough, MA 01752 USA). Rapid on‐site evaluation (ROSE) on air‐dried Diff‐Quik stained smears was performed to assess the adequacy of the collected sample. For all cases, a second pass was performed, and the material was rinsed in 5 mL of formalin to create a cell block. The samples fixed in the hemolytic and preservative solution Cytolyt (Hologic Inc. Marlborough, MA 01752 USA) were centrifuged at 252 *g*, and then the sediment was transferred to the Preservcyt solution (Hologic Inc. Marlborough, MA 01752 USA) to be processed using the T5000 automated processor according to the manufacturer's recommendations. The resulting slides were fixed in 95% ethanol and stained with Papanicolaou to evaluate cellularity. The remaining material was stored in the Preservcyt solution for further investigations, such as HPV‐DNA testing.

### 2.3 HPV‐DNA Test

HPV‐DNA extraction was carried out using Mag—Bind Blood and Tissue DNA HDQ Omega on the automatized platform MGISP‐960 (MGI Tech, Guangodong, China). Real‐time PCR was carried out using the AnyplexTM II HPV28 detection kit (CE‐IVD, Seegene Inc. Seoul, South Korea) on the CFX96TM RT‐PCR System (Bio‐Rad Laboratories Inc. Hercules, California, USA). This test allows to amplify nucleic acids for qualitative detection and HPV genotyping by using specific pairs of primers. The results were interpreted using Seegene Viewer software for Real Time Instrument V3. Twenty‐eight HPV genotypes were investigated, including high‐risk genotypes (16, 18, 31, 33, 35, 39, 45, 51, 52, 56, 58, 59, 66, 68, 69, 73, 82), low‐risk genotypes (6, 11, 40, 42, 43, 44, 54, 61, 70), and probable oncogenic genotypes (26, 53).

## 3 Results

### 3.1 General Features of the Series

The study included 66 cases from 65 patients (37 males and 28 females, M:F ratio 1.32), observed between May 2022 and January 2023, with ages ranging from 22 to 85 years (mean age 61.33 years). Most lesions were located in the parotid gland (63 out of 66 cases, 95.5%), 2 cases (3.0%) in the submandibular gland, and 1 case (1.5%) in the right cheek. On US examination, the nodular lesions were predominantly hypoechoic and heterogeneous, with sizes ranging from 10 to 60 mm.

### 3.2 Cytological Diagnoses

In all cases, findings from FNAC were classified according to the Milan system [7]. The sampling was adequate in all cases. Out of the total, 15 cases (22.7%) were diagnosed as non‐neoplastic, 5 cases (7.6%) as atypia of undetermined significance, 30 cases (45.5%) as benign neoplasms, 7 cases (10.6%) as salivary gland neoplasms of uncertain malignant potential, 2 cases (3.0%) as suspicious for malignancy, and 7 cases (10.6%) as malignant neoplasms. None of the cases were classified as nondiagnostic. Histological control was available in 34 cases. In 15 cases (22.7%), a diagnosis of non‐neoplastic pathology was rendered (Milan System Category II), including 4 cases of sialolithiasis, 2 cases of reactive intraparotid lymph nodes, 3 cases of nonspecific intraglandular cysts, and 6 cases of chronic sialoadenitis. Of the patients diagnosed with intraglandular cysts, two underwent surgical resection, and histopathological examination identified one lymphoepithelial cyst and one keratin cyst. Atypia of undetermined significance (Milan System Category III) was identified in 5 cases (7.6%), typically displaying cytologic features of inflammatory cells, histiocytes, crystalloid formations, and limited clusters of epithelial cells with hyperchromatic nuclei and dense cytoplasm. Histological controls were available in three of these cases, resulting in diagnoses of myoepithelioma, Warthin tumor, and cystadenoma, respectively. A benign neoplasm diagnosis (Milan System Category IVA) was assigned in 30 cases (45.5%), comprising 12 cases of pleomorphic adenoma and 18 cases of Warthin tumor. Histologic controls were available in 19 of these cases, confirming 9 pleomorphic adenomas and 10 Warthin tumors. Seven cases (10.6%) were classified as salivary gland neoplasms of uncertain malignant potential (SUMP; Milan System Category IVB), including 4 cases of oncocytic neoplasm and 3 cases of basal cell neoplasm. Histological controls for these cases revealed 1 Warthin tumor, 1 oncocytoma, 1 basal cell adenoma, and 1 carcinoma ex pleomorphic adenoma. A diagnosis of suspicious for malignancy (Milan System Category V) was rendered in 2 (3.0%) cases, including 1 clear cell neoplasm and 1 B‐cell lymphoproliferative disease. Follow‐up for these patients was unavailable. Finally, a malignant neoplasm diagnosis (Milan System Category VI) was rendered in 7 cases (10.6%), including 3 cases of squamous cell carcinoma (SCC), 2 cases of poorly differentiated carcinoma, 1 case of salivary duct carcinoma, and 1 case of Langerhans cell histiocytosis. Histologic confirmation was available in 5 cases, including 3 SCCs, 1 poorly differentiated carcinoma, and Langerhans cell histiocytosis. The cytological diagnoses according to the Milan system and histological controls are summarized in Table 1.

**TABLE 1:** Cytological diagnosis according to Milan reporting system.

Milan System Category	*N* (%) cases	Cytological diagnosis	*N* cases per type of diagnosis	HPV positive cases	*N* cases histological controls	Histological diagnosis (*N* cases)
II. Non neoplastic	15 (22.7%)	Sialolithiasis	4	0/4	2	Lymphoepithelial cyst Keratin cyst
		Intraparotid lymph node	2	0/2		
		Intraglandular cyst	3	2/3		
		Chronic sialoadenitis	6	0/6		
III. AUS	5 (7.6%)	Inflammatory cells, histiocytes, crystalloid formations, and few epithelial groups with hyperchromatic nuclei and dense cytoplasm	5	0/3	3	Myopepithelioma Warthin tumor Cystadenoma
IVA. Benign neoplasm	30 (45.5%)	Pleomorphic adenoma	12	0/12	19	Pleomorphic adenoma Warthin tumor
		Warthin tumor	18	0/18		
IVB. SUMP	7 (10.6%)	Oncocytic cell neoplasm	4	0/4	4	Warthin Tumor Oncocytoma Basal cell adenoma Carcinoma ex pleomorphic adenoma
		Basal cell neoplasm	3	0/3		
V. Suspicious for malignancy	2 (3.0%)	Clear cell neoplasm	1	0/1	0	
		B‐cell lymphoproliferative disease	1	0/1		
VI. Malignant	7 (10.6%)	Squamous cell carcinoma	3	2/3	6	Squamous cell carcinoma Poorly differentiated carcinoma Langerhans cell histiocytosis
		Poorly differentiated carcinoma	2	1/2		
		Salivary duct carcinoma	1	0/1		
		Langerhans cell histiocytosis	1	0/1		
TOT	66			5/66	34/66	

Abbreviations: AUS: atypia of undetermined significance; SUMP: salivary gland neoplasm of uncertain malignant potential.

### 3.3 HPV‐DNA Test

HPV‐DNA testing was carried out on all samples, yielding adequate results in 65 out of 66 cases (98.5%). In only one case, corresponding to a pleomorphic adenoma, the test was deemed inadequate. Overall, HPV DNA was detected in 5 samples (7.6%) using PCR. Among these, 3 cases were metastatic SCCs, and 2 cases were intraglandular salivary cysts. The most prevalent genotype detected was HPV16, found in 3 out of 5 cases (60.0%). HPV16 was detected in 2 SCCs and in 1 salivary intraglandular cyst. HPV66 was found in 1 (20.0%) salivary intraglandular cyst, and HPV33 was found in the remaining SCC (20.0%). None of the primary salivary gland neoplasms diagnosed via FNAC tested positive for HPV.

### 3.4 HPV‐Positive Cases

**FIGURE 1:**
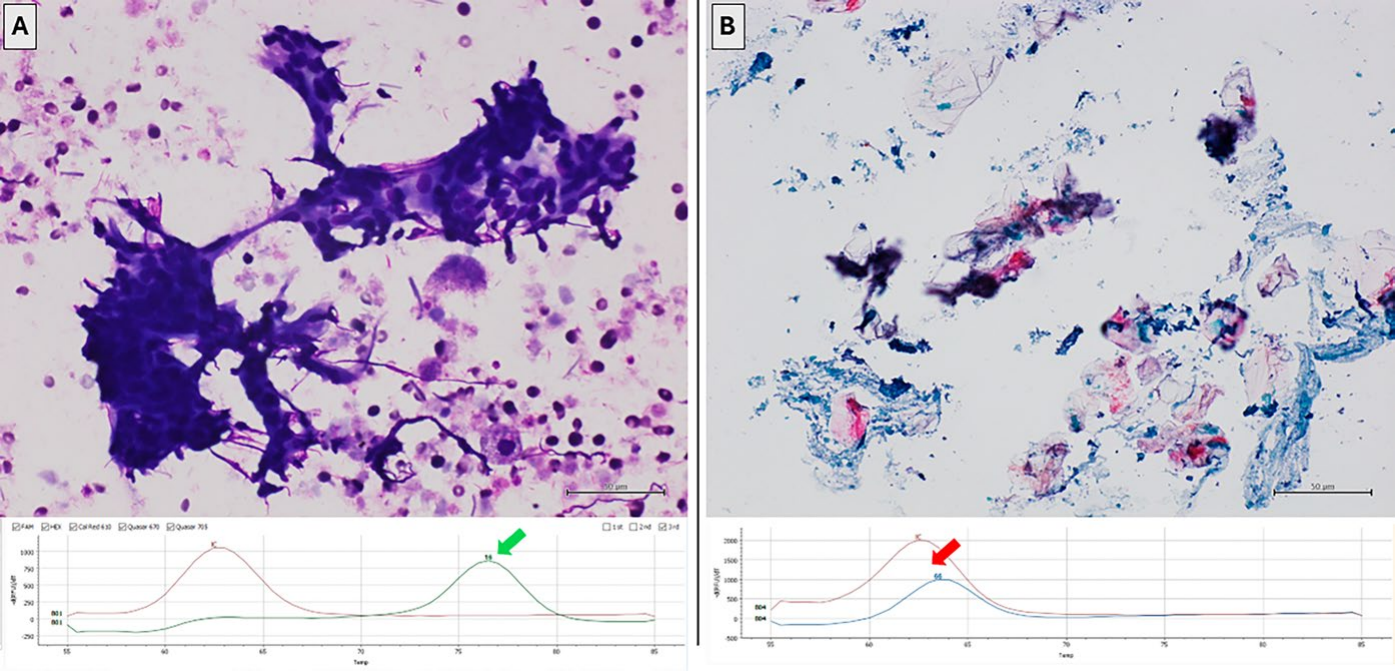
(A) Right parotid HPV16‐positive cyst. Top: US‐guided FNAC showing epithelial cell group characterized by small‐sized cellular elements with a round nucleus and variable quantity of cytoplasm. Distorted chromatin filaments are observed in the clusters. In the protein background, isolated cells with foamy cytoplasm (May Grunwald Giemsa stain, original magnification 400×). Bottom: PCR demonstrating the presence of the HPV16 genotype (green arrow). (B) Right parotid HPV66‐positive cyst. Top: US‐guided FNAC showing proteinaceous background with keratin lamellae (Papanicolaou stain, original magnification 400×). Bottom: PCR demonstrating the presence of the HPV66 genotype (red arrow). [Color figure can be viewed at wileyonlinelibrary.com]

**FIGURE 2:**
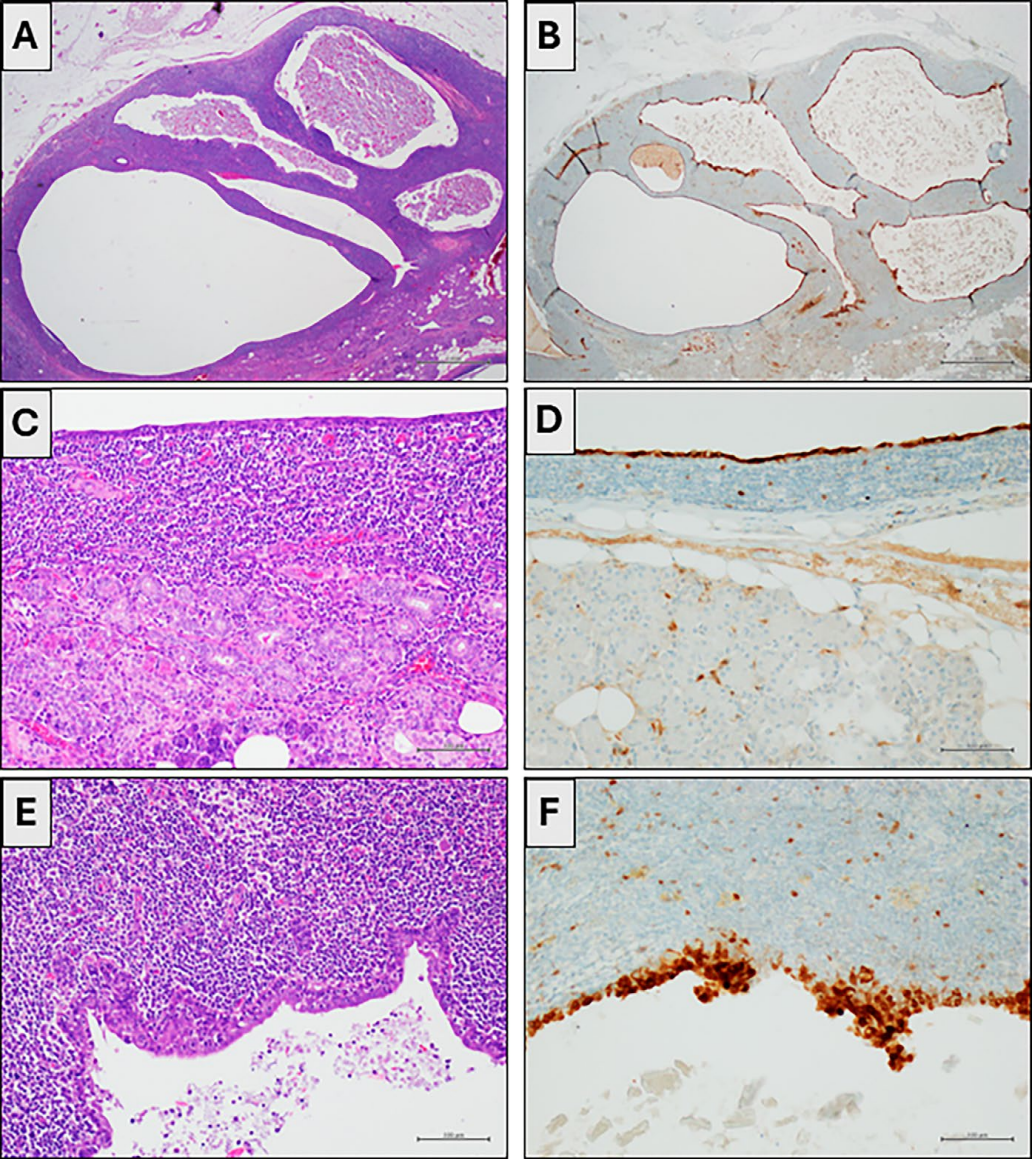
(A) A multilocular cyst with a dense lymphoid cuff. Normal parotid gland parenchyma is observed in the lower right corner (H&E, 4×). (B) p16 positivity (p16 immunostaining, 4×). (C) The cysts are variably lined by squamous cell epithelium. There is an intense infiltrate of lymphocytes in the cyst wall with acinic structures (H&E, 20×). (D) p16 positivity in the cyst with squamous cell epithelium (p16 immunostaining, 20×). (E) Some cysts are lined by columnar epithelium subtended by intense lymphocytic infiltrate (H&E, 20×). (F) p16 positivity is also observed in the cyst with columnar epithelium (p16 immunostaining, 20×). [Color figure can be viewed at wileyonlinelibrary.com]

Concerning the 2 HPV‐positive intraglandular cysts, one patient (HPV 16‐positive) presented with bilateral parotid swelling. On US, both swellings exhibited features of a cystic lesion. FNAC was performed on the two cysts at different times. The first cyst was located at the right parotid and tested positive for HPV16‐DNA (Figure 1). The second cyst, located in the left parotid and morphologically compatible with sialolithiasis, tested negative for HPV‐DNA. Histological control was available for the HPV16‐positive cyst (Figure 2). The lesion was fully excised and submitted in entirety for histopathological examination, revealing a multilocular cyst lined with thin squamous epithelium without cellular atypia nor solid pattern of growth. A dense lymphoid infiltrate was noted within the cyst wall thickening. No cellular atypia or solid epithelial proliferation was observed across all examined sections. Immunohistochemical staining for p16 was diffusely positive in the epithelial lining. A definitive diagnosis of lymphoepithelial cyst was established. HPV‐DNA testing was subsequently performed on formalin‐fixed, paraffin‐embedded tissue from the excised cyst, confirming the presence of HPV16 DNA. Surgical intervention was not pursued for the patient with an HPV66‐positive cyst. Both patients with HPV‐positive cysts underwent further evaluation to rule out pharyngeal malignancies. Medical history was negative for previous pharyngeal lesions. Comprehensive assessments, including ENT examination, ultrasound (US) imaging of the head and neck lymph nodes, and CT scanning of the head and neck, yielded negative results. Follow‐up over 16 and 18 months, respectively, including ENT pharyngeal examination and head/neck US imaging, showed no recurrence or complications in either patient. Concerning the 3 HPV‐positive SCCs, all cases were confirmed as metastases from oropharyngeal carcinoma through biopsy in this district.

## 4 Discussion

The correlation between tumors and viruses, such as HPV, is well‐established for various tumors, including those of the anogenital and head and neck regions. However, for some tumors, this correlation remains unclear. The potential role of HPV in the pathogenesis of salivary gland neoplasms is not well understood, and the existing literature presents conflicting data. Moreover, although previous studies have shown that HPV is present in the saliva of some individuals, it is not clear from which specific HPV reservoir chronic infection arises, allowing for its persistence in the saliva of these subjects. A study by Vageli et al. demonstrated a promising association between HPV and salivary gland lesions, with HPV being detected in nearly 78% of cases [1]. Similarly, Hühns et al. in 2015 found an association in 76% of cases [8]. However, other studies have reported varying degrees of association, ranging from 57.6% to 23.5% of cases [1, 2, 3, 9, 10, 11, 12]. Conversely, studies by Skalova et al. and Haeggblom et al. in 2018 refuted these findings [1, 2, 3]. Skalova et al. [2] did not detect HPV in any of the 55 salivary gland tumors studied, while Haeggblom et al. [3] found HPV 16 positivity in only one case, which was a SCC of the parotid gland. In a few other studies, the presence of the viral genome was demonstrated in an insignificant percentage (ranging from 2.1% to 7.4%) of cases of salivary gland neoplasia [1, 2, 3, 13, 14, 15, 16]. The literature review on the field is detailed in Table 2.

**TABLE 2:** Review of the literature.

Authors, year	*N*. cases	M:F; age range	Sites	Diagnosis	HPV status	HPV‐related neoplasm	Sample type HPV‐detection method
Vageli 2007 [1]	9	7:2; 11–70	Parotid gland	1 oncocytoma, 1 ACC, 1 HG AdenoCA, 1 WT, 2 PA, 1 polymorphous adenoCA, 1 lipoma, 1 lymphoepithelial cyst	7/9 HPV 16 HPV 16–18 HPV 18–6 HR HPV	77.8%	FFPE histopathological material PCR
Boland 2012 [16]	27	22:5; 37–89	6 maxilla, 6 submandibular gland, 1 sublingual gland, 2 palate, 2 sphenoid sinus/nasal, septum, 1 base of tongue, 4 parotid gland, 2 nasal cavity, 1 external auditory canal, 1 lacrimal gland, 1 larynx	AdCC	2/27 HR HPV	7.4%	FFPE histopathological material In situ hybridization
Hafed 2012 [12]	34	12:22; 23–90	Salivary gland	8 PA, 1 myoepithelioma, 6 WT, 7 AdCC, 1 EmyECA, 1 lymphoepithelial CA, 1 lymphoma, 7 MEC, 1 myoepithelial CA, 1 polymorphous adenoCA	8/34 HP16/18	23.5%	FFPE histopathological material In situ hybridization
Descamps 2012 [15]	79	36:44; 15–88	3 submandibular gland, 69 parotid gland, 7 oral cavity	40 PAP, 5 AdCC, 9 MEC, 9 Ca ex‐PA, 6 ACC	4/79 HPV‐16 LR‐HPV	5.1%	FFPE histopathological material PCR
Skalova 2013 [2]	55	NA	Major and minor salivary gland	14 PA, 9 WT, 4 myoepithelioma, 1 oncocytoma, 1 cystadenoma, 1basal cell adenoma, 4 polymorphous adenoCA, 7 cribriform adenoCA of Tongue, 6 AdCC, 2 SDC, 2 MEC, 2 ACC, 1 HCCC, 1 carcinosarcoma ex pleomorphic adenoma	0/55	0.0%	FFPE histopathological material PCR
Teng 2014 [9]	59	NA	Parotid gland	36 PA, 12 WT, 3 adenoma, 1 myoepithelioma, 3 adenoCA, 1 AdCC, 1 MEC, 2 others	34/59 7 LR‐HR HPV 11 mixed HR HPV 16 single LR or HR HPV (were mainly HPV 16 and 18) HPV52 in benign neoplasm	57.6%	FFPE histopathological material PCR and flow‐through hybridization
Hühns 2015 [8]	200	107:93; 11–95	175 parotid gland, 14 submandibular gland, 11 minor salivary gland	17 MEC, 16 AdCC, 10 adenoca NOS, 9 SDC, 7 ACC, 5 Adenoid basal‐cell carcinoma, 13 SCC	19/25 HPV‐16	76.0%	FFPE histopathological material PCR
Qian 2016 [10]	67	23:44; 16–90	17 parotid gland, 10 submandibular gland, 40 minor salivary gland	AdCC	28/67 HPV‐16 HPV‐18 HPV‐11 HPV‐33,59 HPV‐45, 56 HPV‐45	41.8%	FFPE histopathological material PCR
Haeggblom 2018 [3]	107	NA; 47–85	Parotid gland	22 ACC, 19 adenoCA NOS, 13 AdCC, 3 basal cell adenoCA, 4 EmyeCA, 22 MEC, 2 myoepithelial CA, 1 oncocytic CA, 3 poorly differentiated CA, 10 SDC, 1 secretory CA, 7 SCC	1/107 HPV 16	0.93%	FFPE histopathological material PCR
Mohamed 2021 [11]	18	NA	Salivary gland	6 MEC, 6 AdCC, 6 myoepithelial CA	6/18 HPV 16	33.3%	FFPE histopathological material PCR
Gu 2022 [13]	48	NA	Major and minor salivary gland	MEC	1/48 HPV 16	2.1%	FEPE histopathological material NGS‐based tumor analysis
Zupancic 2022 [14]	68	24:44; 13–88 years	22 submandibular gland, 20 parotid gland, 11 oral cavity, 7 nasal and paranasal sinuses, 5 tongue bases/tonsil, 2 lip, 1 nasopharynx	AdCC	3/68 HPV 33 HPV 16	4.4%	FFPE histopathological material PCR

Abbreviations: ACC: acinic cell carcinoma; AdCC: adenoid cystic carcinoma; Ca‐exPA: carcinoma ex‐pleomorphic adenoma; EmyECA: epithelia‐myoepithelial carcinoma; FFPE: formalin‐fixed and paraffin‐embedded; HCCC: hyalinizing clear cell carcinoma; HG AdenoCa: high grade adenocarcinoma; HPV: human papillomavirus; HR HPV: high risk human papillomavirus; LR‐HPV: low risk human papillomavirus; MEC: mucoepidermoid carcinoma; NGS: next generation sequency; PA: pleomophic adenoma; PCR: polymerase chain reaction; SCC: squamous cell carcinoma; SDC: salivary duct carcinoma; WT: Warthin tumor.

Different technologies have been used in different studies to detect HPV, including in situ hybridization (ISH), PCR, and NGS. However, in all studies, molecular research was carried out on formalin‐fixed and paraffin‐embedded histological samples. Our study was conducted on cytological samples obtained by salivary gland lesion FNAC. FNAC specimens obtained from cervical lymph nodes have been used in the past to evaluate the presence of HPV DNA in patients with metastatic oropharyngeal SCC or cancer of unknown origin (CUP) [1, 17, 18]. To the best of our knowledge, cytological samples from FNAC of salivary glands have never been used for this purpose.

The primary salivary gland neoplasms in our series all tested negative for HPV DNA, suggesting that the virus does not play a role in the development of salivary gland neoplasms. These findings are consistent with recent literature, which contradicts earlier evidence suggesting a link between HPV infection and primary salivary gland neoplasms. Variability in detection methods, sample sizes, geographic factors, and the potential for contamination may have contributed to the inconsistent findings reported in the literature. The absence of a clear association between HPV and salivary gland tumors seems plausible, given that the typical HPV‐driven oncogenic process—mediated by viral oncoproteins E6 and E7, which inactivate tumor suppressor proteins p53 and Rb—has not been observed in salivary gland tumors. Additionally, there is no consistent evidence of E6/E7 expression in these neoplasms.

In our series, intraglandular localization from SCC tested positive for HPV, and subsequent analysis revealed metastases originating from SCC in the oropharynx. Oropharyngeal SCC is known to be associated with HPV infection. This data is significant as it could assist in the differential diagnosis between primary SCC of the salivary gland, although rare, and metastatic oropharyngeal carcinoma, as suggested in a previous study [17]. The use of HPV testing on FNAC samples in clinical practice could easily resolve this differential diagnosis. Particularly noteworthy is that the diagnosis of oropharyngeal SCC in our series was performed retrospectively based on the results obtained from FNAC diagnosis of the salivary gland. In two cases of SCC metastases, HPV16 was identified, while HPV33 was detected in the third case. HPV16 is responsible for 85%–96% of oropharyngeal SCC cases. Other high‐risk HPV types—including HPV18, HPV31, HPV33, and HPV35—are less common but still significant contributors to head and neck cancers, particularly in the oropharynx [19]. Notably, HPV33 is identified in approximately 1%–5% of HPV‐positive oropharyngeal SCC cases across studies [1, 20, 21]. Interestingly, HPV33 is also the predominant HPV type associated with HPV‐related multiphenotypic sinonasal carcinoma (HMSC), a rare and distinct form of sinonasal carcinoma [22]. The differential diagnosis between HMSC and SCC may be challenging on cytological samples. HMSC displays a combination of squamous and glandular (epithelial) as well as myoepithelial differentiation, moderate nuclear pleomorphism, which includes a mix of small, round to oval cells with larger, more irregularly shaped cells, cytoplasmic vacuoles, and necrotic material in the background. However, in this instance, the identification of an oropharyngeal primary tumor excluded the possibility of HMSC.

Arguably, the most unexpected finding of this study is the detection of HPV in two salivary intraglandular cysts lined by squamous cell epithelium. We detected HPV16‐DNA in a right parotid gland cyst, which was subsequently excised, and histological examination showed a lymphoepithelial cyst. HPV16‐DNA was also detected on the histological sample, and p16 immunohistochemistry resulted positive. p16 positivity is defined as a strong and diffuse staining of both the nuclei and cytoplasm [23]. p16 immunohistochemistry is often used as a surrogate marker for HPV infection, particularly in HPV‐related cancers and pre‐cancerous lesions, especially where HPV‐DNA testing might not be feasible or readily available [23]. The overexpression of p16, a cyclin‐dependent kinase inhibitor, is associated with the disruption of cell cycle regulation by high‐risk HPV oncoproteins, such as E6 and E7, which inactivate tumor suppressors (p53 and Rb proteins). The differential diagnosis between lymphoepithelial cyst and intraglandular metastasis of SCC was mainly based on the absence of cytological atypia, mitoses, and infiltrative patterns. However, clinical‐pathological correlation is often necessary for the differential diagnosis. Indeed, the patient was investigated to exclude the presence of pharyngeal neoplasms; no previous pharyngeal lesion was present in anamnesis, and the follow‐up was uneventful. The identification of HPV‐DNA in salivary gland cysts is an unreported data that raises at least two significant questions: first, it prompts inquiry into whether HPV is the primary etiological factor of these lesions, or if HPV merely colonizes such cystic lesions within the salivary glands, potentially serving as a reservoir for the virus. In the latter scenario, HPV‐positive salivary gland cysts could be considered reservoirs for chronic reinfection in certain individuals, facilitating both the spread of the virus within the population and its reinfection within the oropharynx of affected individuals. Moreover, it would be intriguing to explore how the identification of the viral genome could be leveraged to gain clinical–diagnostic advantages. Several studies in the literature have already delved into the presence of HPV in saliva and attempted to elucidate its role. For instance, Kai Dun Tang et al. reported a case where the first SCC of the oropharynx was diagnosed very early (at stage T1N0M0) through the screening of HPV 16 in saliva [4]. The search for the viral genome in saliva has also been validated by other studies, such as those by Qureishi A. et al. [5] and Borsetto et al. [24].

In conclusion, our study analyzed FNAC samples from salivary gland lesions to explore a potential association with HPV infection. This is the first study using DNA from cytological samples for this purpose. We found evidence of HPV presence in two intraglandular cysts lined by squamous cell epithelium. The demonstration of HPV in salivary cysts has never been reported previously and therefore requires further investigation to clarify its biological significance and clinical value. We also found HPV in three SCC located in the parotid gland, diagnosed as oropharyngeal SCC metastases. However, HPV testing yielded negative results in all primary salivary gland neoplasms, failing to confirm the potential involvement of HPV infection in the pathogenesis of salivary gland tumors.

## Author Contributions

**Immacolata Cozzolino:** conceptualization, investigation, writing – original draft, methodology, writing – review and editing, data curation. **Andrea Ronchi:** conceptualization, writing – original draft, writing – review and editing, data curation. **Marco Montella:** writing – original draft, data curation. **Raffaella Ruggiero:** investigation, methodology. **Rosaria Cappiello:** investigation, methodology. **Giovanni Savarese:** investigation, methodology. **Giuseppe Colella:** data curation. **Renato Franco:** writing – review and editing, supervision.

## Conflicts of Interest

The authors declare no conflicts of interest.

## Data Availability

The data that support the findings of this study are available on request from the corresponding author. The data are not publicly available due to privacy or ethical restrictions.

## References

[1] D. P. Vageli , G. Sourvinos , M. Ioannou , G. K. Koukoulis , and D. A. Spandidos , “High‐Risk Human Papillomavirus (HPV) in Parotid Lesions,” International Journal of Biological Markers 22 (2007): 239–244. 18161653 10.1177/172460080702200401

[2] A. Skálová , J. Kašpírková , P. Andrle , L. Hostička , and T. Vaneček , “Human Papillomaviruses are not Involved in the Etiopathogenesis of Salivary Gland Tumors,” Ceskoslovenská Patologie 49, no. 2 (2013): 72–75. 23641711

[3] L. Haeggblom , R. G. Ursu , L. Mirzaie , et al., “No Evidence for Human Papillomavirus Having a Causal Role in Salivary Gland Tumors,” Diagnostic Pathology 13, no. 1 (2018): 44, 10.1186/s13000-018-0721-0. 30021645 PMC6052678

[4] K. D. Tang , S. Vasani , T. Taheri , et al., “An Occult HPV‐Driven Oropharyngeal Squamous Cell Carcinoma Discovered Through a Saliva Test,” Frontiers in Oncology 10 (2020): 408, 10.3389/fonc.2020.00408. 32296641 PMC7136454

[5] A. Qureishi , M. Ali , L. Fraser , K. A. Shah , H. Møller , and S. Winter , “Saliva Testing for Human Papilloma Virus in Oropharyngeal Squamous Cell Carcinoma: A Diagnostic Accuracy Study,” Clinical Otolaryngology 43, no. 1 (2018): 151–157. 28620984 10.1111/coa.12917

[6] K. K. S. Rieth , S. R. Gill , A. A. Lott‐Limbach , et al., “Prevalence of High‐Risk Human Papillomavirus in Tonsil Tissue in Healthy Adults and Colocalization in Biofilm of Tonsillar Crypts,” JAMA Otolaryngology–Head & Neck Surgery 144, no. 3 (2018): 231–237. 29372248 10.1001/jamaoto.2017.2916 PMC5885877

[7] E. D. Rossi , Z. Baloch , and G. Barkan , “Second edition of the Milan System for Reporting Salivary Gland Cytopathology: Refining the Role of Salivary Gland FNA,” Cancer Cytopathology 132, no. 1 (2024): 10–21. 37971077 10.1002/cncy.22753

[8] M. Hühns , G. Simm , A. Erbersdobler , and A. Zimpfer , “HPV Infection, but Not EBV or HHV‐8 Infection, Is Associated with Salivary Gland Tumours,” BioMed Research International 2015 (2015): 829349. 26618178 10.1155/2015/829349 PMC4651650

[9] W. Q. Teng , X. P. Chen , X. C. Xue , et al., “Distribution of 37 Human Papillomavirus Types in Parotid Gland Tumor Tissues,” Oncology Letters 7, no. 3 (2014): 834–838. 24527091 10.3892/ol.2013.1770 PMC3919866

[10] X. Qian , A. M. Kaufmann , C. Chen , et al., “Prevalence and Associated Survival of High‐Risk HPV‐Related Adenoid Cystic Carcinoma of the Salivary Glands,” International Journal of Oncology 49, no. 2 (2016): 803–811. 27279281 10.3892/ijo.2016.3563

[11] F. E. Mohamed , L. N. Aldayem , M. A. Hemaida , et al., “Molecular Detection of Human Papillomavirus‐16 Among Sudanese Patients Diagnosed with Squamous cell Carcinoma and Salivary Gland Carcinoma,” BMC Research Notes 14, no. 1 (2021): 56. 33563329 10.1186/s13104-021-05471-5 PMC7871554

[12] L. Hafed , H. Farag , O. Shaker , and D. El‐Rouby , “Is Human Papilloma Virus Associated with Salivary Gland Neoplasms? An In Situ‐Hybridization Study,” Archives of Oral Biology 57, no. 9 (2012): 1194–1199. 22542162 10.1016/j.archoralbio.2012.03.009

[13] W. Gu , A. Bhangale , M. E. Heft Neal , et al., “Analysis of Human Papilloma Virus Content and Integration in Mucoepidermoid Carcinoma,” Viruses 14, no. 11 (2022): 2353. 36366450 10.3390/v14112353 PMC9698779

[14] M. Zupancic , S. Holzhauser , and L. Cheng , “Analysis of Human Papillomavirus (HPV) and Polyomaviruses (HPyVs) in Adenoid Cystic Carcinoma (AdCC) of the Head and Neck Region Reveals Three HPV‐Positive Cases with Adenoid Cystic‐like Features,” Viruses 14, no. 5 (2022): 1040. 35632780 10.3390/v14051040 PMC9144058

[15] G. Descamps , A. Duray , A. Rodriguez , et al., “Detection and Quantification of Human Papillomavirus in Benign and Malignant Parotid Lesions,” Anticancer Research 32, no. 9 (2012): 3929–3932. 22993339

[16] J. M. Boland , E. D. McPhail , J. J. García , J. E. Lewis , and D. J. Schembri‐Wismayer , “Detection of human papilloma virus and p16 expression in high‐grade adenoid cystic carcinoma of the head and neck,” Modern Pathology 25, no. 4 (2012): 529–536. 22157933 10.1038/modpathol.2011.186

[17] M. Montella , R. Ruggiero , G. Savarese , et al., “Parotid Squamous Cell Carcinoma Metastases: Application of Human Papillomavirus‐DNA Test on Liquid‐Based Cytology to Recognize Oropharyngeal Origin of the Neoplasm,” Diagnostic Cytopathology 52, no. 9 (2024): E187–E193. 38676309 10.1002/dc.25334

[18] F. Rollo , M. G. Dona' , R. Pellini , et al., “Cytology and Direct Human Papillomavirus Testing on Fine Needle Aspirates from Cervical Lymph Node Metastases of Patients with Oropharyngeal Squamous Cell Carcinoma or Occult Primary,” Cytopathology 29, no. 5 (2018): 449–454. 29873841 10.1111/cyt.12581

[19] M. Lechner , J. Liu , L. Masterson , and T. R. Fenton , “HPV‐Associated Oropharyngeal Cancer: Epidemiology, Molecular Biology and Clinical Management,” Nature Reviews Clinical Oncology 19, no. 5 (2022): 306–327. 10.1038/s41571-022-00603-7 PMC8805140 35105976

[20] H. Mehanna , T. Beech , T. Nicholson , et al., “Prevalence of Human Papillomavirus in Oropharyngeal and Nonoropharyngeal head and Neck Cancer‐‐Systematic Review and Meta‐Analysis of Trends by Time and Region,” Head & Neck 35, no. 5 (2013): 747–755. 22267298 10.1002/hed.22015

[21] G. M. Clifford , S. Gallus , R. Herrero , et al., “Worldwide Distribution of Human Papillomavirus Types in Cytologically Normal Women in the International Agency for Research on Cancer HPV Prevalence Surveys: A Pooled Analysis,” Lancet 366, no. 9490 (2005): 991–998. 16168781 10.1016/S0140-6736(05)67069-9

[22] L. D. R. Thompson and J. A. Bishop , “Update from the 5th Edition of the World Health Organization Classification of Head and Neck Tumors: Nasal Cavity, Paranasal Sinuses and Skull Base,” Head and Neck Pathology 16, no. 1 (2022): 1–18. 35312976 10.1007/s12105-021-01406-5 PMC9018924

[23] A. D. Singhi and W. H. Westra , “Comparison of Human Papillomavirus In Situ Hybridization and p16 Immunohistochemistry in the Detection of Human Papillomavirus‐Associated Head and Neck Cancer Based on a Prospective Clinical Experience,” Cancer 116, no. 9 (2010): 2166–2173. 20186832 10.1002/cncr.25033

[24] D. Borsetto , J. Cheng , K. Payne , et al., “Surveillance of HPV‐Positive Head and Neck Squamous Cell Carcinoma with Circulating and Salivary DNA Biomarkers,” Critical Reviews in Oncogenesis 23, no. 3–4 (2018): 235–245. 30311577 10.1615/CritRevOncog.2018027689

